# Identifying Changes in Peripheral Lymphocyte Subpopulations in Adult Onset Type 1 Diabetes

**DOI:** 10.3389/fimmu.2021.784110

**Published:** 2021-12-06

**Authors:** Aina Teniente-Serra, Eduarda Pizarro, Bibiana Quirant-Sánchez, Marco A. Fernández, Marta Vives-Pi, Eva M. Martinez-Caceres

**Affiliations:** ^1^ Immunology Division, Clinical Laboratory MetroNord (LCMN), Germans Trias i Pujol University Hospital and Research Institute (IGTP), Barcelona, Spain; ^2^ Department of Cell Biology, Physiology and Immunology, Universitat Autònoma de Barcelona, Spain; ^3^ Endocrinology Department, Hospital de Mataró, Barcelona, Spain; ^4^ Flow Cytometry Facility, Germans Trias i Pujol Research Institute (IGTP), Barcelona, Spain

**Keywords:** immunomonitoring, lymphocyte subpopulations, flow cytometry, type 1 diabetes, onset, adult, follow-up

## Abstract

T- and B-lymphocytes play an important role in the pathogenesis of type 1 diabetes (T1D), a chronic disease caused by the autoimmune destruction of the insulin-producing cells in the pancreatic islets. Flow cytometry allows their characterization in peripheral blood, letting to investigate changes in cellular subpopulations that can provide insights in T1D pathophysiology. With this purpose, CD4^+^ and CD8^+^ T cells (including naïve, central memory, effector memory and terminally differentiated effector (TEMRA), Th17 and Tregs) and B cells subsets (naïve, unswitched memory, switched memory and transitional B cells) were analysed in peripheral blood of adult T1D patients at disease onset and after ≥2 years using multiparametric flow cytometry. Here we report changes in the percentage of early and late effector memory CD4^+^ and CD8^+^ T cells as well as of naïve subsets, regulatory T cells and transitional B cells in peripheral blood of adult patients at onset of T1D when compared with HD. After 2 years follow-up these changes were maintained. Also, we found a decrease in percentage of Th17 and numbers of T cells with baseline. In order to identify potential biomarkers of disease, ROC curves were performed being late EM CD4 T cell subset the most promising candidate. In conclusion, the observed changes in the percentage and/or absolute number of lymphocyte subpopulations of adult T1D patients support the hypothesis that effector cells migrate to the pancreas and this autoimmune process perseveres along the disease. Moreover, multiparametric flow allows to identify those subsets with potential to be considered biomarkers of disease.

## Introduction

In type 1 diabetes (T1D) there is a destruction of insulin producing cells as a consequence of an autoimmune response in which lymphocyte subpopulations are crucial. Antigen-specific CD8^+^ T lymphocytes play an important role in the autoimmune response in T1D at the pancreas (1, 2). Due to its inaccessibility, few studies have been performed analysing islet insulitis in the target tissue. Even so, it has been proved that at the onset of the disease resident memory CD8^+^ T cells are infiltrating the gland (3, 4). Moreover, an increase of Th17 cells and a decrease in number and functionality of Tregs has been found in pancreatic lymph nodes, supporting their role in the pathogenesis of T1D (5).

A large number of existing studies have examined alterations in peripheral lymphocyte subpopulations, although most of them have been performed in paediatric patients with T1D (6–11). In general, it has been reported an increase of Th1 and Th17 cells in peripheral blood, whereas Tregs cells are impaired, supporting their involvement in the pathogenesis of T1D (12–15).

In contrast to paediatric studies, the research of lymphocyte subpopulations in adulthood T1D remains limited. It was reported in the onset of the disease a predominance of an IFNγ-producing response (16). Additionally, higher amounts of Th17 cells were found in adult newly diagnosed patients (17). To our knowledge, only Apostolopoulou et al. performed a longitudinal study regarding the correlation of peripheral leukocyte subpopulations and their correlation with metabolic parameters (18).

Recent advances in the development of multiparametric flow cytometry have made possible an exhaustive and detailed characterisation of lymphocyte subsets in whole blood or isolated peripheral blood mononuclear cells (PBMC) of healthy donors and patients (19–22) and has been shown as a powerful tool for immunomonitoring of autoimmune diseases.

In order to properly address this question, we performed an exhaustive flow cytometric analysis in whole blood. We therefore analysed what are lymphocyte subpopulations like when found in peripheral blood in adulthood at onset of T1D and investigated whether these changes were maintained at established disease. The aim of this work is to define which changes detected in peripheral lymphocytes using flow cytometry at the onset of T1D could be potential biomarkers of disease in order to identify those individuals at risk to develop the autoimmune response.

## Materials and Methods

### Study Subjects and Design

Patients with T1D were recruited from the outpatient clinic of the Endocrinology department of Hospital de Mataró (Mataró, Spain). Thirty-six adult patients with newly (<6 months) diagnosed T1D were enrolled in the study. The mean age was 28.86 years ± 8 and 51.43% (n=18) of them were women (**Supplementary Table 1**). Patients had a diagnosis of T1D according to ADA and WHO criteria and were positive for at least one autoantibody associated with T1D (anti-GAD, anti-IA2, anti-ICA and anti-insulin). Mean time after diagnosis was 3 months ± 1.35. Six patients were also analysed at long-standing stage (> 2 years) of T1D (4.35 years ± 1.35). Fresh blood samples of 40 healthy donors (HD) from the Blood Bank of Catalonia (Spain) were used as controls. The mean age was 42.85 years ± 10.83 and 57.5% (n=23) of them were women. The study was approved by the local Ethics Committee and written informed consent was obtained from all T1D patients and HD.

### Sample Collection

Blood samples of 10 mL from T1D patients and control samples were collected in EDTA tubes (BD Biosciences, San Jose, CA, USA). Blood was analysed in the first 4 hours after venepuncture, being viability of cells in all cases >99%.

### Flow Cytometry and Analysis

As previously described (23), whole blood samples (100µl) were incubated with the appropriate amounts of monoclonal antibodies for 20 minutes at room temperature and protected from the light. After erythrocyte lysis, samples were washed and a total of 100,000 lymphocytes were acquired on a FACSCantoII flow cytometer (BD Biosciences).

Lymphocyte subpopulations were defined according to the markers specified in **Table 1** and using different combinations of the following monoclonal antibodies anti-: CD3-AmCyan, CD4-V450, CCR6-phycoerythrin (PE), CCR4-AlexaFluor 647, CD4- allophycocyanin (APC)-H7, CD8-V450, CD45RA-fluorescein isothiocyanate (FITC), CCR7-PE-Cy7, CD27 PerCP-Cy5.5, CD31-AlexaFluor 647, CD19-PerCP-Cy5.5, CD27-APC, CD24-FITC, CD38-PE, CD19 V500, CD3-V450, IgM-PerCP-Cy5.5, IgD FITC, CD21-PE, CD45-FITC, CD4-PERCP-Cy5.5, CCR4-PE-Cy7, CD127-A647, CD45RO-APC-H7, CD25-PE, HLA-DR-V500 (BD Biosciences) and PTK7-PE (Milteny Biotec, Bergisch Gladbach, Germany). The desired lymphocyte subpopulations were gated using doublet discrimination and forward and side scatter, and their relative percentages were determined. The gating strategy for lymphocyte subsets was previously described (23 and **Supplementary Figures 1**–**7**). The flow cytometry data were analysed using FACSDiva software (BD Biosciences).

**Table 1:** Lymphocyte subpopulations and phenotype analyzed.

Lymphocyte subpopulation	Phenotype
**T cell subsets**	
** Naive**	CD3+CD4+CD27+CCR7+CD45RA+
	CD3+CD8+CD27+CCR7+CD45RA+
** Central memory (T_CM_)**	CD3+CD4+CD27+CCR7+CD45RA-
	CD3+CD8+CD27+CCR7+CD45RA-
** Early effector memory (T_early EM)_**	CD3+CD4+CD27+CCR7-CD45RA-
	CD3+CD8+CD27+CCR7-CD45RA-
** Late effector memory (T_late EM_)**	CD3+CD4+CD27-CCR7-CD45RA-
	CD3+CD8+CD27-CCR7-CD45RA-
** Terminally differenciated effector cells (T_EMRA_)**	CD3+CD4+CD27-CCR7-CD45RA+
	CD3+CD8+CD27-CCR7-CD45RA+
** DN lymphocytes**	CD3+CD4-CD8-
**DPs lymphocytes (CD4+CD8+)**	CD3+CD4+CD8+CD27+CCR7+CD45RA+
	CD3+CD4+CD8+CD27+CCR7+CD45RA-
	CD3+CD4+CD8+CD27+CCR7-CD45RA-
	CD3+CD4+CD8+CD27-CCR7-CD45RA-
	CD3+CD4+CD8+CD27-CCR7-CD45RA+
** Th17**	CD4+CCR7-CCR4+CCR6+
** Memory Treg**	CD4+CD127lowCD25+CCR4+CD45RO+
** Activated Treg**	CD4+CD127lowCD25+CCR4+CD45RO+HLADR+
**B cell subsets**	
** Naive**	CD19+CD27-IgD+IgM+
** Unswitched memory**	CD19+CD27+IgD+IgM+
** IgM-only memory**	CD19+CD27+IgD-IgM+
** Switched memory**	CD19+CD27+IgD-IgM-
** Transitional B cells**	CD19+CD24hiCD38hiCD27-
** Immature B cell**	CD19+CD27-IgD+IgM+CD21low

Fluorescence minus one (FMO) controls were used to define CCR7 expression on Th17 cells and PTK7 expression on recent thymic emigrants (RTEs).

### Quantification of Absolute Cell Numbers

Quantification of lymphocyte numbers procedure was previously described (23). Briefly, 25 µl of peripheral blood samples were incubated with CD45-PerCP (Beckton Dickinson (BD) Biosciences, San José, CA, USA) for 15 minutes at room temperature and protected from the light. Erythrocytes were removed using 450 µl lysis buffer (BD FACS™ Lysing Solution, BD Biosciences). 25 µl of PerfectCount beads (Cytognos SL, Salamanca, Spain) were added to each sample and acquired on the same flow cytometer. The number of total lymphocytes was expressed in cells/µl.

Percentage and absolute counts (number of cells per µl) were analysed for all subpopulations defined in **Table 1**. Absolute counts were calculated as follows: (%subset/100) x counts of main subpopulations.

### Statistical Analysis

For clinical characteristics (mean ± standard deviation) a descriptive statistical analysis of the main variables was performed. Differences between two groups (HD vs onset patients, or onset vs long-course patients) were analysed using the non-parametric Mann-Whitney U test. The results were expressed as medians (interquartile range). Two-tailed P values <0.05 were considered statistically significant. Receiver operating characteristic (ROC) curves were performed to evaluate the predictive value of percentages and absolute number of each lymphocyte subpopulation. All statistical analyses were performed using the Statistical Package for Social Sciences (SPSS/Windows version 24.0; SPSS Inc, Chicago, IL, USA) and the software program GraphPad Prism (8.0 version; GraphPad, La Jolla; CA, USA).

## Results

### Lymphocyte Subsets Are Altered at the Early Stages of T1D

**Figure 1:**
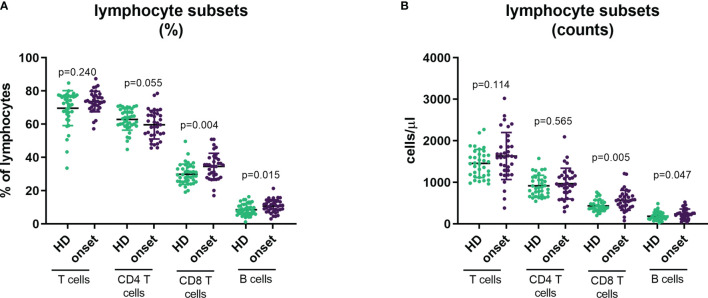
Changes in main lymphocyte subpopulations at onset of T1D. Percentage **(A)** and absolute counts **(B)** of main lymphocyte subpopulations (CD3+, CD4+, CD8+ and CD19+ lymphocytes) in peripheral blood of patients with T1D at onset of the disease and HD. T1D=type 1 diabetes; HD=, healthy donor.

To provide a comprehensive landscape of lymphocyte subpopulations of peripheral blood we performed 5 different panels of surface flow cytometry staining to analyse 33 T-cell and B-cell subsets (both in percentage and absolute counts). First, we examined the percentage and absolute counts of main lymphocyte subpopulations CD3^+^ cells (T cells), CD4^+^ T cells, CD8^+^ T cells, CD19^+^ cells (B cells), as well as double-positive T cells for CD4 and CD8 (DP T cells) and double-negative T cells for CD4 and CD8 (DN T cells). We found an increase in the percentage and absolute counts of CD8^+^ T cells (p=0.004 and p=0.005, respectively) and in the percentage of total B cells (p=0.015), whereas a low decrease in the percentage of CD4^+^ T cells (p=0.013) was also observed (**Figure 1**). Interestingly, as we previously described in other autoimmune diseases, as Graves’ disease (24), patients with T1D had higher percentage and numbers of DP T cells (p=0.039; **Table 2**).

**Table 2:** Changes in lymphocyte subpopulations in peripheral blood of healthy donors (HD) vs patients at onset of T1D.

	Parent	Percentage (%) Healthy donors		Percentage (%) onset of T1D		p	Counts (cells/µl) Healthy donors		Counts (cells/µl) onset of T1D		p
**CD3+ lymphocytes**	Lymphocytes	72.5	[66.1-76.9]	73.6	[70.02-77.2]	ns	1449	[1163-1674]	1605	[1284-1861]	ns
** CD4+ lymphocytes**	T cells	63.3	[59.5-68.9]	60.1	[53.65-66.6]	ns	878	[698-1123]	939	[757-1154]	ns
** CD4 naïve**	T CD4	31.3	[26.26-40.20]	41.3	[29.6-48.2]	**0.013**	265	[201-365]	384	[231-481]	**0.037**
** CD4 central memory**	T CD4	30.3	[24.47-33.25]	32	[25.3-39.55]	ns	243	[206-295]	338	[232-395]	**0.042**
** CD4 early EM**	T CD4	21.3	[17.61-28.93]	15	[9.7-20.2]	**<0.001**	202	[132-251]	146	[106-192]	**0.008**
** CD4 late EM**	T CD4	7.7	[4.85-10.39]	3.2	[1.8-3.9]	**<0.001**	68	[39-90]	28	[19-44]	**<0.001**
** CD4 TEMRA**	T CD4	0.75	[0.12-2.25]	0.1	[0-0.9]	**0.007**	7	[1-18]	1	[0-9]	**0.024**
** RTE**	T CD4 naïve	2.8	[2.1-4.3]	2.9	[2.3-3.95]	ns	7	[5-15]	11	[6-21]	ns
** Th17**	T CD4	4.5	[3.43-6.79]	4.9	[3.7-5.87]	ns	47	[30-59]	45	[31-55]	ns
** regulatory T cells**	T CD4	5.4	[3.80-6.17]	3.5	[2.62-4.67]	**<0.001**	39	[29-49]	35	[26-47]	ns
** activated Tregs**	T CD4	1.9	[1.50-2.4]	1	[0.72-1.37]	**<0.001**	15	[11-19]	10	[7-15]	**0.004**
**CD8+ lymphocytes**	T cells	28.8	[25.5-34.3]	34.5	[28.17-39.45]	**0.004**	404	[339-516]	554	[406-670]	**0.005**
** CD8 naïve**	T CD8	26.3	[18.81-41.6]	37.6	[27.65-51.75]	**0.002**	106	[69-149]	218	[126-302]	**<0.001**
** CD8 central memory**	T CD8	8.6	[4.22-12.52]	6.3	[3.65-11.1]	ns	35	[17-55]	36	[20-49]	ns
** CD8 early EM**	T CD8	28.9	[21.3-31.92]	23.8	[17.4-29.55]	ns	124	[85-149]	123	[85-210]	ns
** CD8 late EM**	T CD8	8.3	[5.6-10.99]	4.2	[2.15-9.35]	**0.005**	34	[15-50]	21	[10-52]	ns
** CD8 TEMRA**	T CD8	15.7	[5.97-21.96]	6.7	[2-17.85]	**0.025**	56	[19-125]	34	[8-95]	ns
**CD4+CD8+ (DPs)**	T cells	0.8	[0.4-1.1]	1	[0.57-1.55]	**0.039**	9	[6-15]	16	[10-25]	**0.01**
** CD45RA^+^CCR7^+^CD27^+^**	T DPs	11.1	[6.7-18]	18.1	[10.05-23.12]	**0.043**	1.32	[0.72-2.43]	2.83	[1.84-3.65]	**0.008**
** CD45RA^-^CCR7^+^CD27^+^**	T DPs	23.1	[15.2-32.42]	19.6	[11.3-35.77]	ns	0.93	[0.60-1.32]	0.79	[0.45-1.43]	ns
** CD45RA^-^CCR7^-^CD27^+^**	T DPs	29.4	[21.77-42.7]	20.6	[13.4-27.67]	**0.009**	1.18	[0.85-1.66]	0.82	[0.54-1.11]	**0.015**
** CD45RA^-^CCR7^-^CD27^-^**	T DPs	7.7	[5.3-11.3]	7.3	[4.62-12.15]	ns	0.31	[0.21-0.50]	0.29	[0.18-0.49]	ns
** CD45RA^+^CCR7^-^CD27^-^**	T DPs	2.9	[1.25-6.5]	3.2	[0.925-12.82]	ns	0.11	[0.04-0.25]	0.13	[0.04-0.51]	ns
**CD19+ cells**	Lymphocytes	8.2	[6.05-10.77]	11	[8.15-13.65]	**0.015**	164	[114-247]	231	[144-340]	**0.047**
** Transitional B cells**	CD19	8.5	[5.61-10.74]	4.5	[2.9-6.95]	**<0.001**	11	[8-25]	11	[6.5-15]	ns
** T1 Transitional B cells**	CD19	2.8	[1.87-3.71]	1.4	[1-2.5]	**<0.001**	4	[3-8]	4	[1.5-5.5]	ns
** T2 Transitional B cells**	CD19	5.5	[3.89-7.19]	3.1	[1.85-4.55]	**<0.001**	8	[5-16]	6	[3.5-10.5]	ns
** Naive B cells**	CD19	61	[45.4-72.17]	67.1	[54.95-76.15]	ns	95	[57-148]	218	[134-318]	**<0.001**
** Immature B cells**	CD19	0.7	[0.3-1]	0.3	[0.2-0.7]	ns	0.9	[0.4-1.4]	0.79	[0.43-3.07]	ns
** Unswitched memory B cells**	CD19	13.6	[9.47-22.77]	10.9	[6.95-12.6]	ns	19	[13-32]	44.15	[22.46-55.49]	**0.031**
** IgM memory B cells**	CD19	1.6	[1.15-2.22]	2.4	[1.45-3.3]	**0.016**	2.35	[1.57-3.15]	8.84	[4.35-13.81]	**<0.001**
** Switched memory B cells**	CD19	15	[10.92-22.57]	13.6	[9.55-18.7]	ns	21	[15-32]	45.81	[25.28-75.72]	**<0.001**

Data expressed as median (interquartile range). Statistical differences calculated from Mann-Whitney test. P values <0.05 considered statically significant (displayed in bold). ns, no significant. HD, healthy donors; T1D, type 1 diabetes; EM, effector memory; RTE, recent thymic emigrants.

### Naïve T Cells Are Increased at the Onset of T1D Whereas There Is a Decrease in Effector Memory Subsets

**Figure 2:**
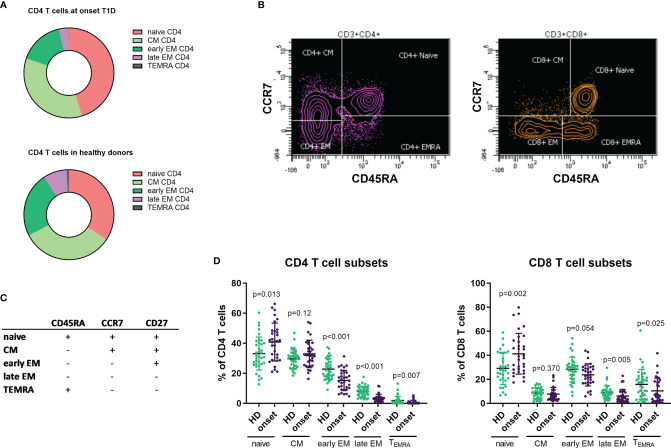
Changes in T CD4 and CD8 subpopulations at onset of T1D. Representation of percentage of T CD4 subsets in patients with T1D at onset of the disease and HD **(A)**. Example of gating strategies for analysis of naïve, CM, EM and EMRA subsets in CD4 and CD8 T cells **(B)**. Markers used to define T cell subsets in CD4 and CD8 cells **(C)**. Percentages of CD4 and CD8 T cell subsets in patients with T1D at onset of the disease compared with HD **(D)**. T1D, type 1 diabetes; HD, healthy donor, CM, central memory, EM, effector memory, EMRA, terminally reverted effector memory.

T cell lymphocytes can be characterized based on the expression in their surface of CD45RA, CCR7 and CD27 in naïve, central memory (CM), early effector memory (early EM), late effector memory (late EM) and effector (EMRA) subsets (**Figures 2B, C**). In the analysis of these subsets in CD4^+^ T cells we observed an increase in the percentage and absolute counts in the naïve subpopulation (p=0.013 and p=0.037) while percentages and absolute counts of early and late EM and EMRA subpopulations were decreased (percentage of early EM p<0.001, late EM p<0.001 and EMRA p=0.007 and counts of early EM p=0.008, late EM p<0.001 and EMRA p=0.024; **Figures 2A, D** and **Table 2**). No changes were found in CM subset.

When we performed the same analysis on CD8^+^ T cells, we also detected an increase in the percentage and counts of the naïve subpopulation (p=0.002 and p<0.001, respectively) and a decrease in the percentages in both late EM and EMRA subsets (p=0.005 and p=0.025, respectively), but no differences were found in their absolute counts (**Figures 2A, D** and **Table 2**).

### At the Onset of T1D There Is a Decrease in Treg Subpopulations

**Figure 3:**
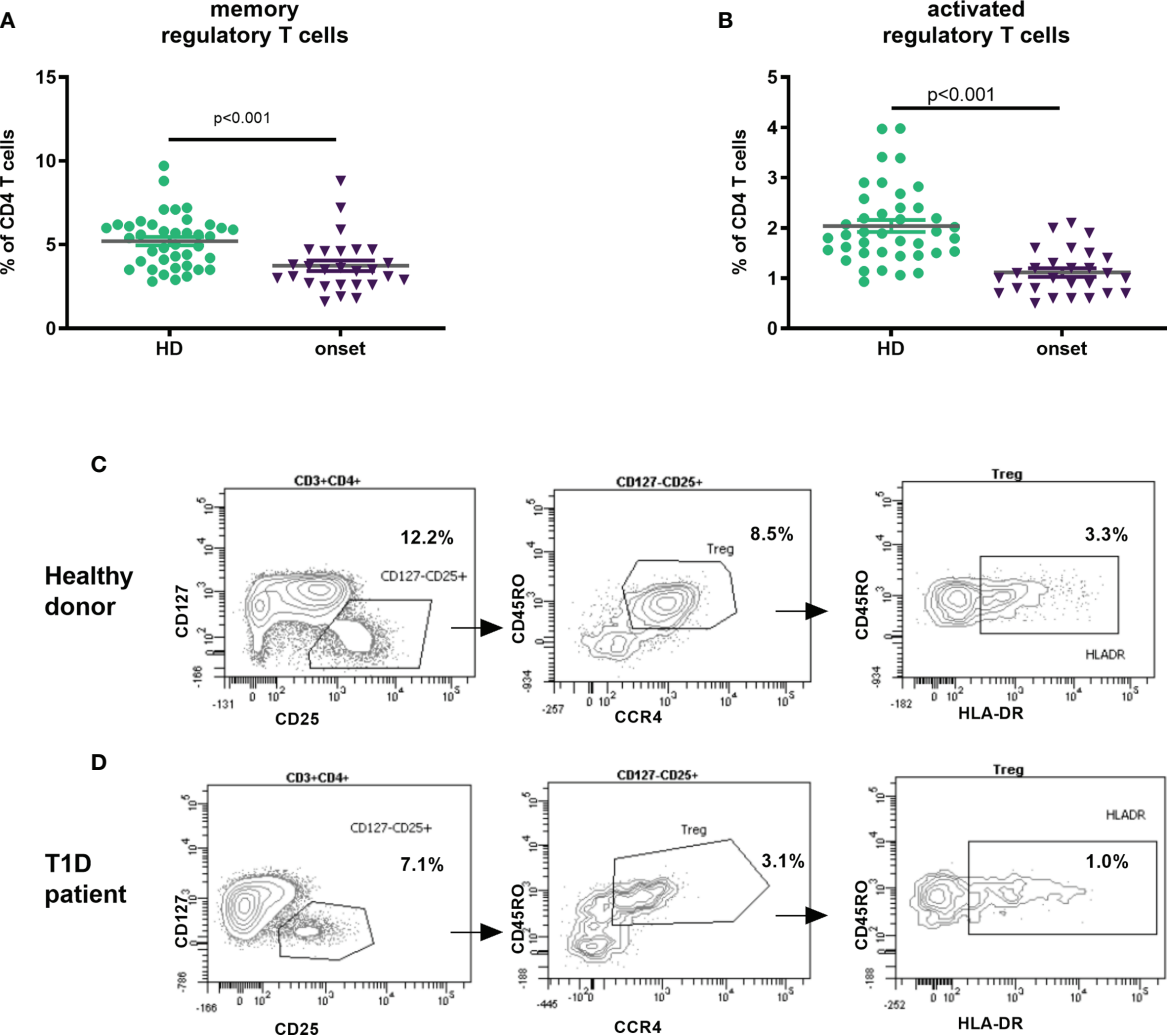
Changes in Treg subpopulations at onset of T1D. Percentage of memory regulatory T cells defined as CD3^+^CD4^+^CD25^hi^CD127^-/low^CD45RO^+^CCR4^+^ **(A)** and activated Tregs CD3^+^CD4^+^CD25^hi^CD127^-/low^CD45RO^+^CCR4^+^HLA-DR^+^ **(B)** in peripheral blood of patients with T1D at onset of the disease and HD. Example of gating Treg subsets (from FSC/SSC; CD45^+^ cells and CD3^+^CD4^+^ cells) in a healthy donor **(C)** and a T1D patient **(D)**. T1D, type 1 diabetes; HD, healthy donor.

An important lymphocyte type in the regulation of autoimmune response is Treg cell. We analysed this subset based of surface markers expression according to HIP-C project (20, 22), defining memory Tregs as CD3^+^CD4^+^CD25^hi^CD127^-/low^CD45RO^+^CCR4^+^ and activated Tregs those expressing HLA-DR. We found that adult patients at T1D onset displayed lower percentage of memory Treg cells (p<0.001, **Figures 3A, C, D**) as well as in the percentage and absolute counts of activated Treg (p<0.001 and p=0.004) in peripheral blood when compared with HD (**Figure 3B** and **Table 2**).

### B Transitional Cells Are Decreased Whereas IgM-only B Cells Are Increased at the Onset of the Disease

**Figure 4:**
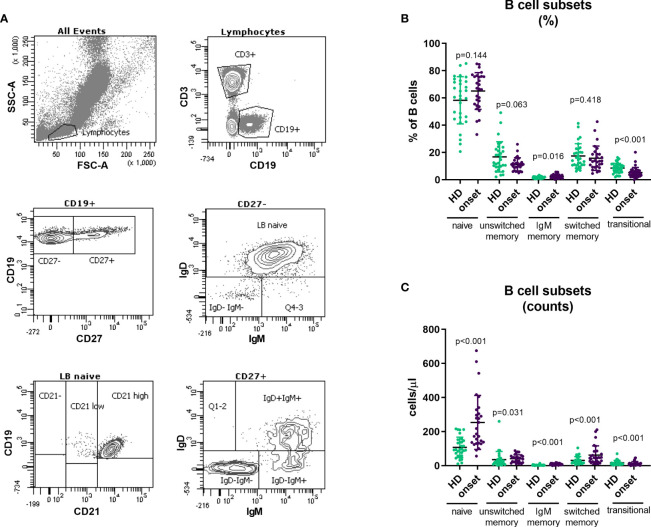
Changes in B cell subpopulations at onset of T1D. Gating strategy for the analysis of B cell subsets **(A)**. Percentage **(B)** and absolute counts **(C)** of B cell subpopulations in peripheral blood of patients with T1D at onset of the disease and HD. T1D, type 1 diabetes; HD, healthy donor.

B cell subpopulations in peripheral blood were analysed according to the expression on their surface of CD27, IgD, IgM, CD24 and CD38 (**Figure 4A**). Adult patients at onset of T1D had higher percentages of IgM-only memory B cells (p=0.016; **Figure 4B**) and higher numbers of B cell subsets due to the higher number of total B cells described above (**Figure 4C** and **Table 2**). Interestingly, we found a decrease in transitional B cells in percentage (p<0.001) when compared with HD (**Figure 4C** and **Table 2**).

### Naïve Double Positive CD4 and CD8 T Cells Are Increased at Onset of T1D

**Figure 5:**
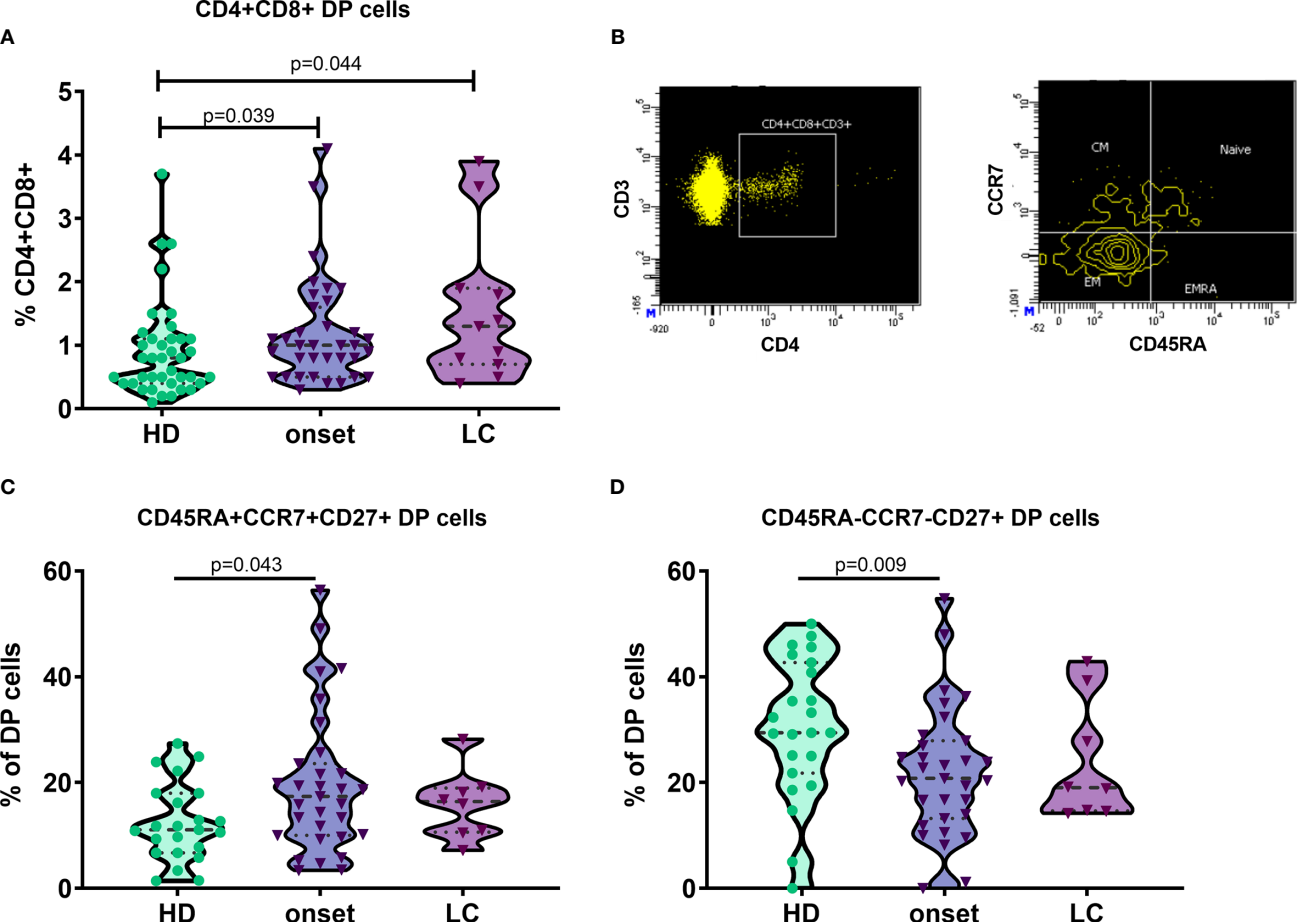
Naïve double positive CD4 and CD8 T cells are increased at onset of T1D. Percentage of double positive (DP) CD4 and CD8 T cells in patients with T1D at onset and at established disease and HD **(A)**. Example of gating strategy for the analysis of CD45RA and CCR7 expression in DP T cells. DP CD4 and CD8 T cells are gated from singlets, FSC/SSC lymphocytes, CD3+ T cells, CD4+ T cells and CD8+ plots **(B)**. Percentage of DP subsets in cells in patients with T1D at onset and at established disease and HD **(C, D)**. T1D, type 1 diabetes; HD, healthy donor; LC, long-course.

As described in our previous works (24, 25), patients with autoimmune diseases show an increase of DP T cells in peripheral blood. When we analysed this subset, we found an increase in T1D patients when compared with HD. In contrast with our results in Graves’ disease patients (24), deeply analysis of this subpopulation showed an increase in percentage and counts of DP cells with a naïve phenotype (CD45RA^+^CCR7^+^CD27^+^; p=0.043 and p=0.008) and a decrease in percentage and counts of DP T cells with an early EM phenotype (CD45RA^-^CCR7^-^CD27^+^; p=0.009 and p=0.015) at the onset of T1D when compared with HD (**Figure 5**).

### Changes in Lymphocyte Subpopulations in Patients at Established Disease

**Figure 6:**
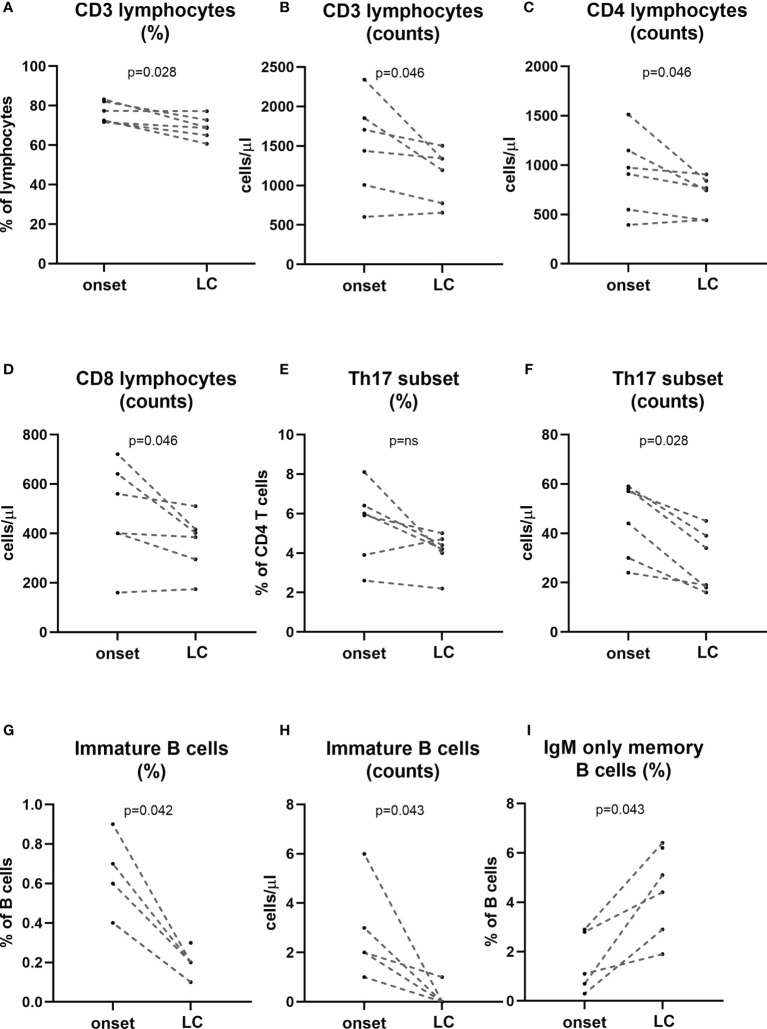
Changes in lymphocyte subpopulations at established disease when compared with onset. A subgroup of patients (n=6) was followed-up at established disease (>2 years of evolution) and compared their own timepoint onset of the disease. Percentage and counts of CD3 **(A, B)**, counts of CD4 and CD8 T cells **(C, D)**, percentage and counts of Th17 cells **(E, F)**, percentage and counts of immature B cells **(G, H)** and percentage of IgM-only memory B cells **(I)** at onset and long-course are represented for each patient. LC, long-course. (p < 0.05 significative; Mann-Whitney test).

In a subgroup of patients, we performed a follow-up analysis at established disease (between 2 and 7 years after diagnosis) and we compared their lymphocyte subpopulations at the onset of the disease with those at established disease. When regarding main lymphocyte subpopulations we found a decrease in the percentage of T cells (p=0.028) as well as a decrease in the absolute counts of total T cells, CD4 and CD8 T cells (p=0.046; **Figure 6**). Interestingly a tendency in the percentage and a statistically significant decrease in the numbers of Th17 subset was observed at established disease when compared with the onset of T1D (p=0.028). Regarding B cell subsets, we found an increase in the percentages of IgM-only memory B cells (p=0.043) and a decrease in the percentage and absolute counts of immature B cells (p=0.042 and p=0.043, respectively; **Figure 6**).

### Peripheral Lymphocyte Subpopulations as Candidate Biomarkers in the Diagnosis of T1D

**Figure 7:**
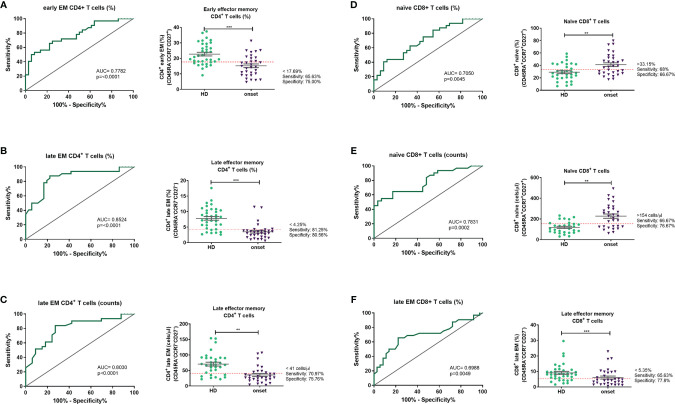
Peripheral lymphocyte subpopulations as candidate biomarkers. Receiver operating characteristic (ROC) curves were performed to evaluate the predictive value of percentages and absolute number of each lymphocyte subpopulation. The ROC curves were selected according to their p value and their AUC. ROC curves and a representation of each subpopulation and the putative cut-off established with the ROC curve are represented for percentages of early EM CD4 T cells **(A)**, percentages and counts of late EM CD4 T cells **(B, C)**, percentage and counts of naïve CD8 T cells **(D, E)** and percentages of late EM CD8 T cells **(F)**. EM, effector memory). **p < 0.01; ***p < 0.001.

Finally, regarding the changes in minor lymphocyte subpopulations detected in peripheral blood from adult patients at the onset of T1D, we performed ROC curves to evaluate these changes as potential future biomarkers to identify prediabetic individuals as well as to allow the proper stratification of patients, the identification of patients with a better glycaemic prognosis, and the discovery of optimal checkpoints for immune interventions. With the results from ROC curves (**Figure 7**), the best candidates to be biomarkers are the percentage and absolute counts of late EM CD4^+^ T cells with a sensitivity and specificity of 81.25% and 80.56%; 70.97% and 75.76% respectively. Concerning CD8^+^ T cells, the percentage of late EM is the best candidate as a biomarker with a sensitivity of 72% and a specificity of 77.8%.

## Discussion

In order to define changes that could be putative biomarkers of disease we performed an extensive whole blood multi-parametric flow cytometry analysis of T and B cell compartments in patients with adult-onset T1D and at established disease. We found an increase of naïve CD4^+^ and CD8^+^, of DP T cells and IgM-only B cells and a decrease of Treg and transitional B cells at onset of the disease. At established disease there is a decrease of Th17 cells and immature B cells, whereas IgM-only B cells increase when compared to onset. Percentage and absolute counts of late EM CD4 T cells are the best candidates to be studied as potential biomarkers of disease.

Previous studies described an increase of Th1 and Th17 cells (mainly at disease onset) and a decrease of Treg subsets (5, 13). Our results are in line with these findings, showing that at the onset of the disease there is an increase of total CD8^+^ T cells and a decrease of total CD4^+^ T cells in peripheral blood, and a bias towards a decrease of memory and effector T cells, which could be infiltrating the pancreatic tissue and perpetuating the autoimmune response. In fact, the presence of tissue resident memory CD8 T cells in insulitic lesions has been demonstrated by Kuric et al. in biopsies of adult patients at onset of the disease (3). We can also confirm that there is a decrease on the percentage of memory Treg cells in peripheral blood at the onset of disease. Disfunction of Treg cells has been associated to autoimmunity in a broad spectrum of autoimmune diseases. In T1D it has been described a decrease of this subset in peripheral blood although there is some controversy in the literature published (10, 26, 27), probably due to variability in the methodology used and in the characteristics of the patients included. Moreover, Ferraro et al. described a decrease in the frequency and functionality of Treg in pancreatic-draining lymph nodes (5). In the same study, authors found an expansion of Th17 cells in pancreatic-draining lymph nodes. In this context, although we didn’t find an increase in the percentage of Th17 cells at the onset of the disease when compared to HD, we showed that in the long-term disease this subset decreased when compared with disease onset. These results enforce the role of Th17 in the early stages of the disease, concurring with the moment with highest level of tissue damage. Moreover, a decrease in the percentage of Th17 in the long-term disease has also been reported in the second year of progression of the disease in pediatric patients (8).

The role of B cells in the initiation and progression of the disease is crucial (28, 29). Here we found a decrease in transitional B cells. Previous published results regarding this subpopulation were not conclusive: whereas Habib et al. (30) found an increase in peripheral blood, Thompson et al. (7) did not show differences. Interestingly, transitional B cells phenotype [defined as CD19^+^CD27^-^CD24^hi^CD38^hi^ (31)] can be included in the IL-10 producing regulatory B subset CD24^hi^CD38^hi^ described by Blair et al. (32). Previous studies found a decrease of this regulatory B subset in T1D patients (33–35) suggesting that the lower frequencies of transitional B cells found in the present study could be related to an impairment in their regulatory properties. Moreover, here we show that patients at onset of the disease have higher percentages of naïve and IgM-only memory B cells, supporting that B cells can play a role in the pathogenesis of the disease, although more studies focusing on it are lacking.

When comparing our results to those in children at onset of T1D, a similar pattern in T cell subsets was found (8): a decrease of memory Tregs and increase of activated Tregs, decrease of Th17 at established disease and a decrease in CD4 and CD8 effector subsets. Interestingly, regarding B cells, no changes either in transitional or naïve and IgM-only memory B cells were observed in children at onset of disease.

It has been postulated that DP cells arise as a result of the failure of maturation of DP thymocytes that do not undergo negative selection and constitute a population of recent thymic emigrants (RTEs) in periphery. Alternatively, DP T cells may result from the expansion in the periphery of mature cells that re-express either CD4 or CD8 (36–38). Last years, our group has been working in elucidate if there is an increase of RTEs in peripheral blood of patients with organ-specific autoimmune diseases. Previous results in patients with Graves’ disease showed an increase of DP T cells in peripheral blood whereas in the thyroid gland an increase of TREC levels was found, suggesting an influx of T cells recently egressed from the thymus into the thyroid (25). However, these results were not confirmed in a second study (24). These discrepancies could be explained by differences in the duration of the disease between both groups of patients, being longer in those with expanded DP T cells. Interestingly, in the present study in adult-onset T1D there was an increase in peripheral blood of DP T cells with a naïve phenotype whereas DP T cells with an early EM phenotype are decreased, when compared with HD. These findings highly suggest the role of a failure in central tolerance mechanisms in the pathogenesis of T1D. However, it should be taken in account that inflammatory and metabolic alterations in T1D can influence the increase of DP T cells at the onset of the disease. For example, it has been described that episodes of hyperglycemia impair phagocytic function (39). So, it is reasonable to speculate that inflammation and hyperglycemia can alter thymic function in diabetes, as reported in ageing (40) and type 2 diabetes (41).

In a subgroup of patients analysed at onset of the disease and again at established disease (more than 2 years after the diagnosis of T1D), a decrease of Th17 cells was found, suggesting that this subset plays a role in the early stages of the disease. Furthermore, immature B cells decrease, and IgM-only memory B cells increase at established disease enforcing the role of B cells in the pathogenesis of the disease.

Until now, autoantibodies against pancreatic islet are the only immunological biomarkers used to define the risk to develop T1D (42). Interestingly, previous studies with antigen-specific cells and in miRNAs in CD4 subsets pointed T cell subsets potential markers of early autoimmune response (43). From all these studies and with the aim to find putative biomarkers of the lymphocyte subpopulations analysed we performed ROC curves. Here we found that, of the 33 subsets analysed, late EM CD4 T cells are the best candidate to be used as biomarkers of T1D. Future research should assess the potential of these subsets as putative biomarkers of developing T1D in those people at risk.

One of the limitations of the study is that this is a descriptive study, and it is difficult to interpret if some of the changes in cell subpopulations are biologically relevant. Functional studies would be necessary to address this. Even though, the presented results identify some changes that may be relevant as putative surrogate markers in the immunomonitoring of the disease.

In conclusion, multiparametric flow cytometry in peripheral blood adult-onset T1D pointed to an important role of minor lymphocyte subpopulations of effector T cells, but also B cells, in the pathogenesis of the disease being impaired those subsets with regulatory characteristics as Tregs and transitional B cells. Most of these changes are maintained at established disease. More studies focusing on late EM CD4 T cells as a potential tool as putative biomarker of disease should be performed.

## Data Availability Statement

The raw data supporting the conclusions of this article will be made available by the authors, without undue reservation.

## Ethics Statement

The studies involving human participants were reviewed and approved by Comitè d’ètica de la investigació amb medicaments, Hospital Universitari Germans Trias i Pujol. The patients/participants provided their written informed consent to participate in this study.

## Author Contributions

AT-S designed the research, performed most of the experiments, analyzed, and interpreted the results, and drafted the manuscript. EP participated in the design of the study, recruitment of patients, interpretation of data and helped in writing the manuscript. BQ-S participated in the interpretation of data and helped in writing the manuscript. MF participated in the design and analysis of flow cytometry experiments. MV-P revised the manuscript. EM-C conceptualized and designed the study, supervised the research and revised the manuscript. All authors contributed to the article and approved the submitted version.

## Funding

The authors are members of a consolidated group (2017SGR103) as recognized by the Agency for Management of University and Research Grants (AGAUR) of the Generalitat of Catalonia. This work has been partially funded by PI20/01313 integrated in the Plan Nacional de I+D+I and co-supported by the ISCIII-Subdirección General de Evaluación and the Fondo Europeo de Desarrollo Regional (FEDER).

## Conflict of Interest

MV-P holds a patent that relate to immunotherapy for T1D and is co-founder of Ahead Therapeutics S.L.

The remaining authors declare that the research was conducted in the absence of any commercial or financial relationships that could be construed as a potential conflict of interest.

## Publisher’s Note

All claims expressed in this article are solely those of the authors and do not necessarily represent those of their affiliated organizations, or those of the publisher, the editors and the reviewers. Any product that may be evaluated in this article, or claim that may be made by its manufacturer, is not guaranteed or endorsed by the publisher.
