# Supplementary figures and images for: Identifying Changes in Peripheral Lymphocyte Subpopulations in Adult Onset Type 1 Diabetes
Source: Front Immunol. 2021 Dec 6;12:784110. doi: 10.3389/fimmu.2021.784110 (PMC8685245; doi:10.3389/fimmu.2021.784110)

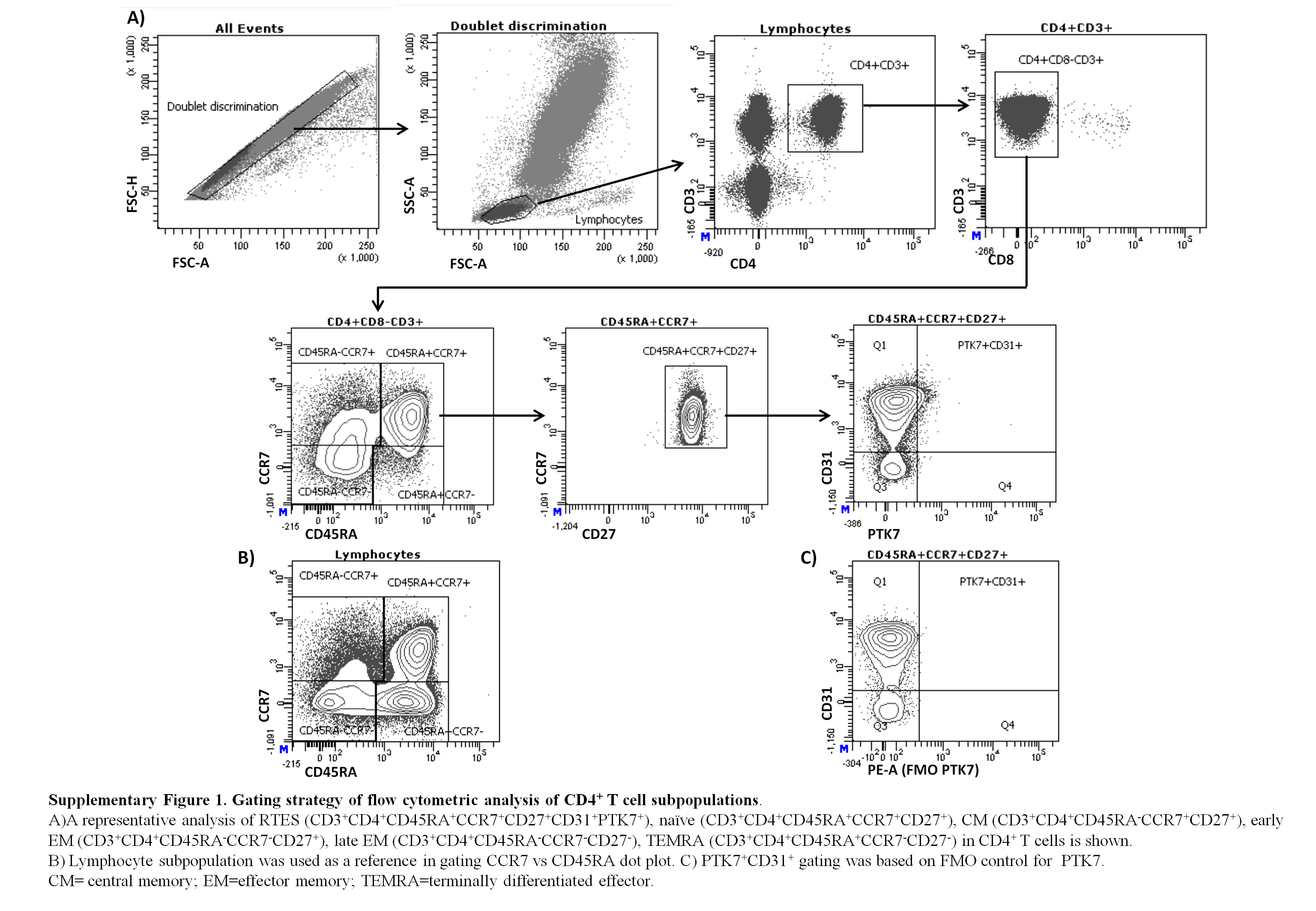

Supplement: Supplementary file 1 [file Image_1.tif]

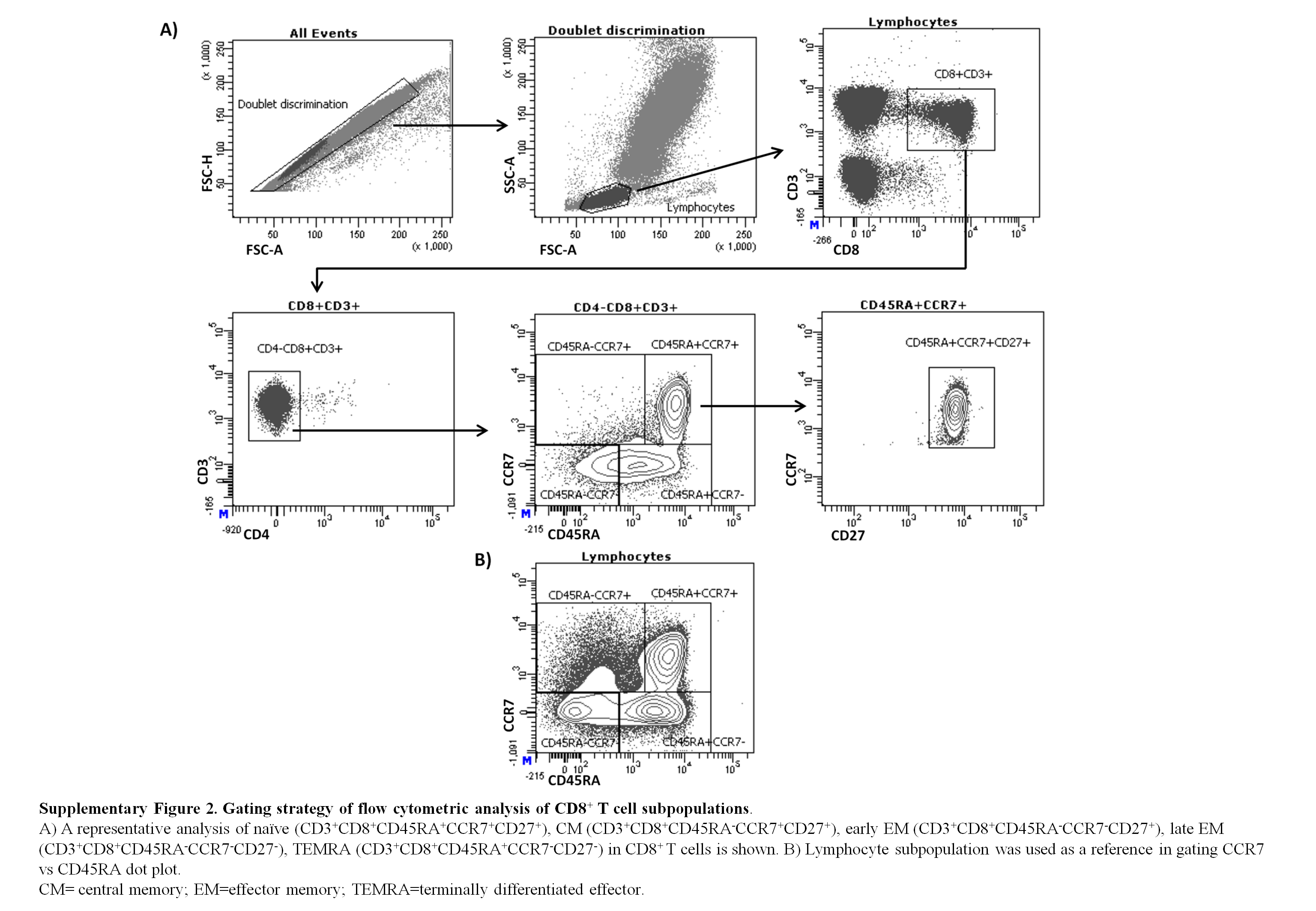

Supplement: Supplementary file 2 [file Image_2.tif]

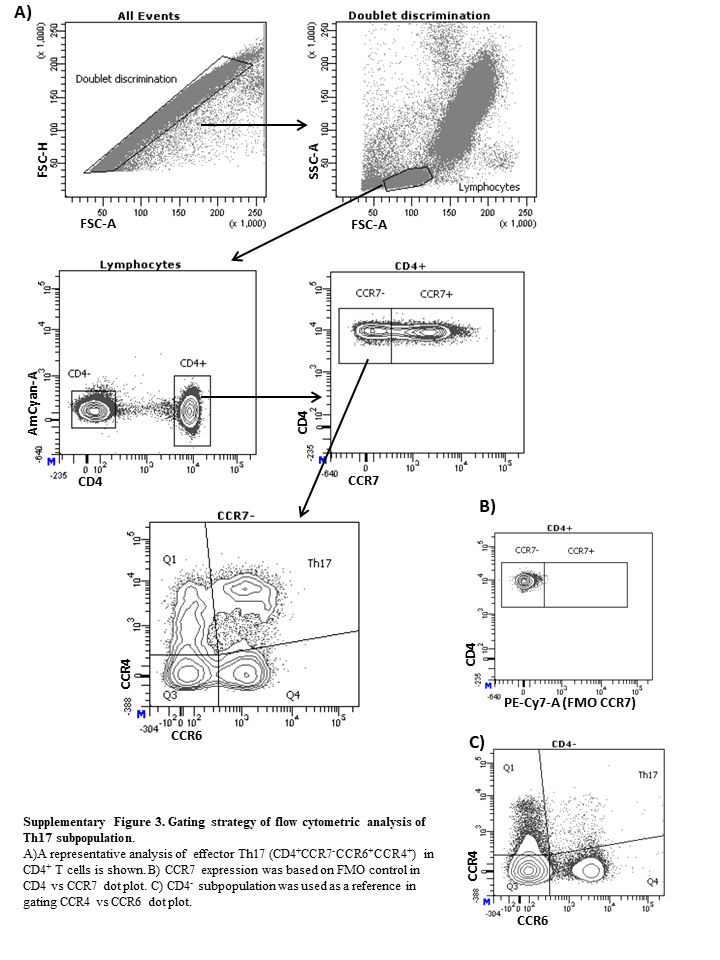

Supplement: Supplementary file 3 [file Image_3.tif]

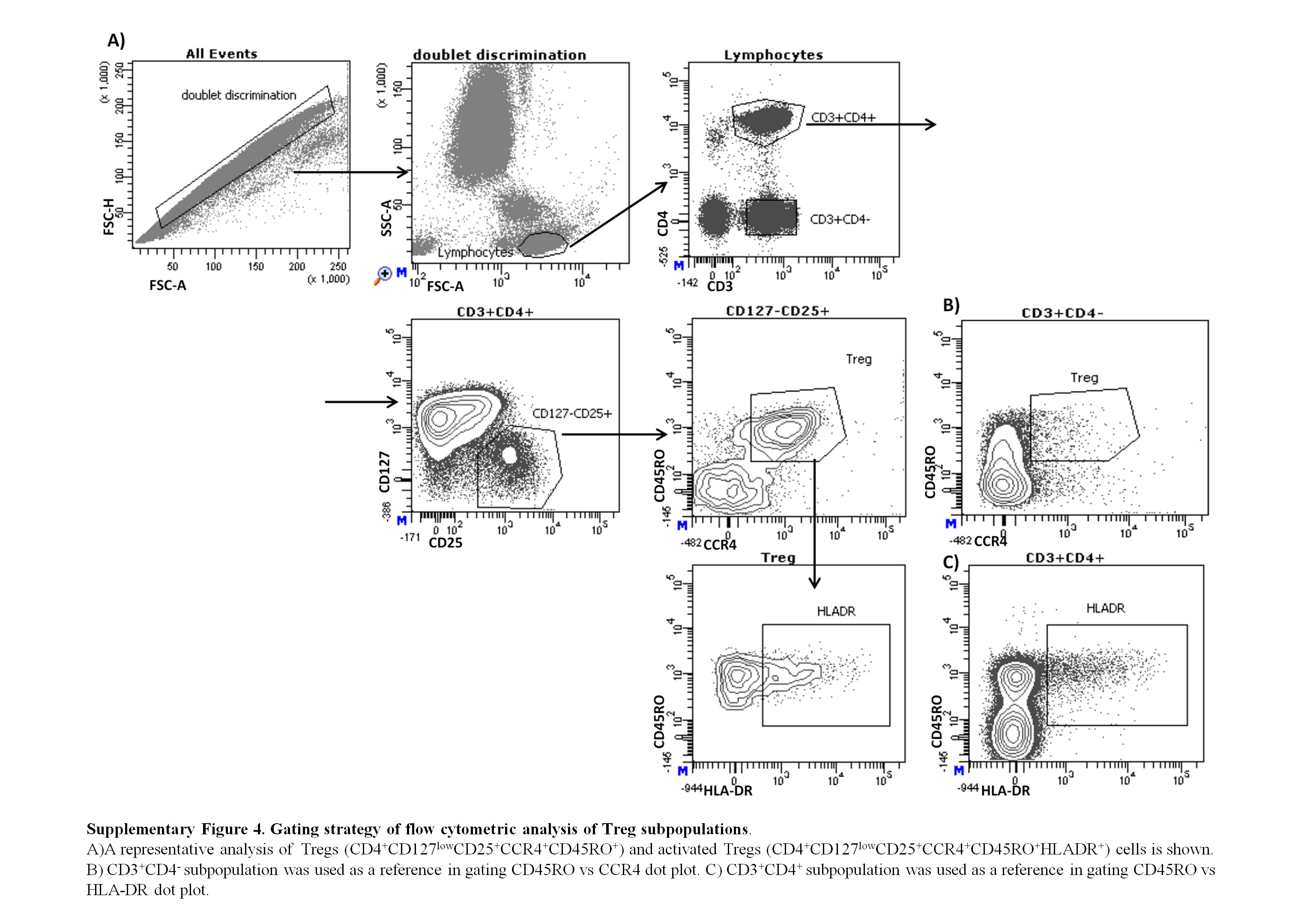

Supplement: Supplementary file 4 [file Image_4.tif]

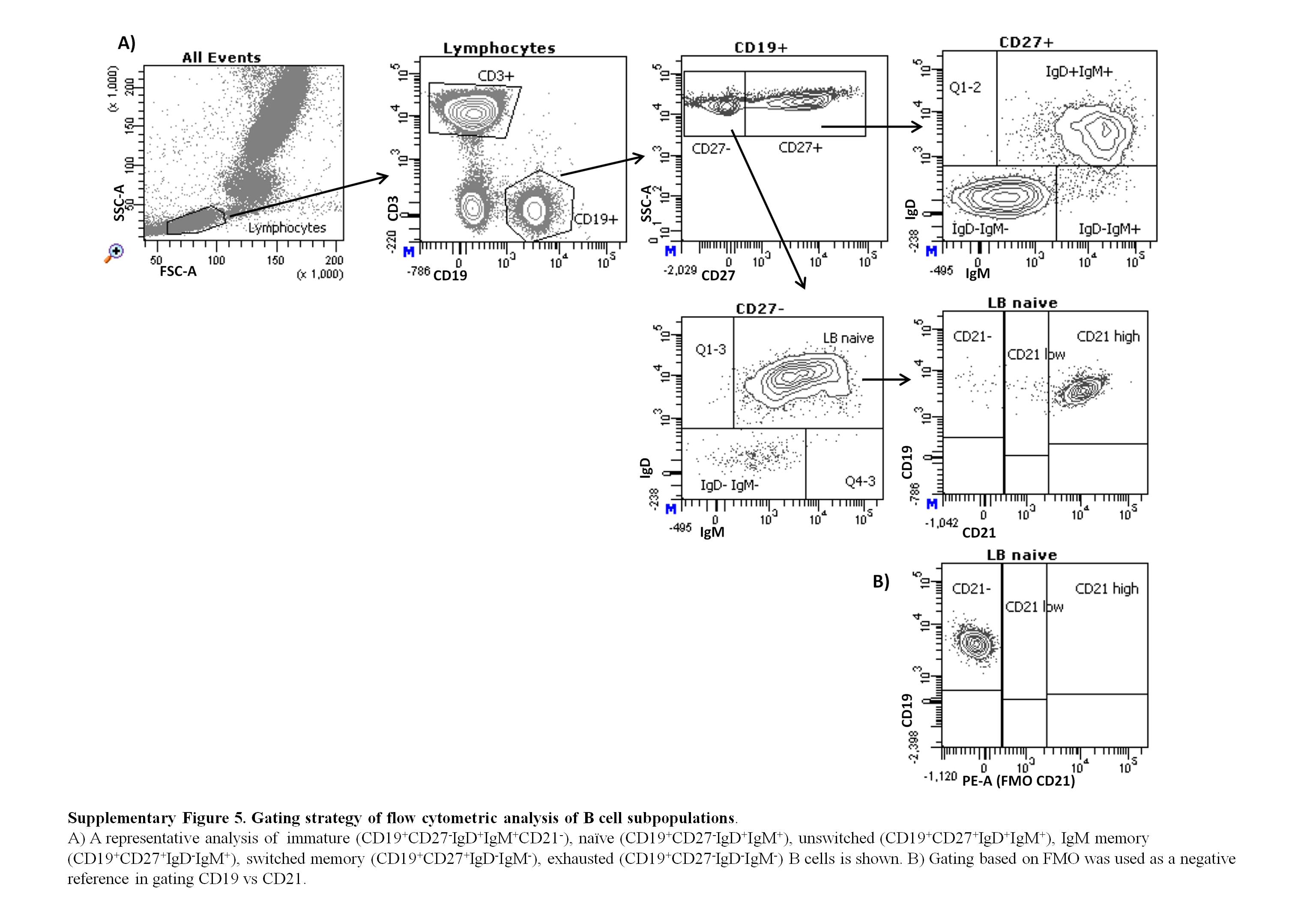

Supplement: Supplementary file 5 [file Image_5.tif]

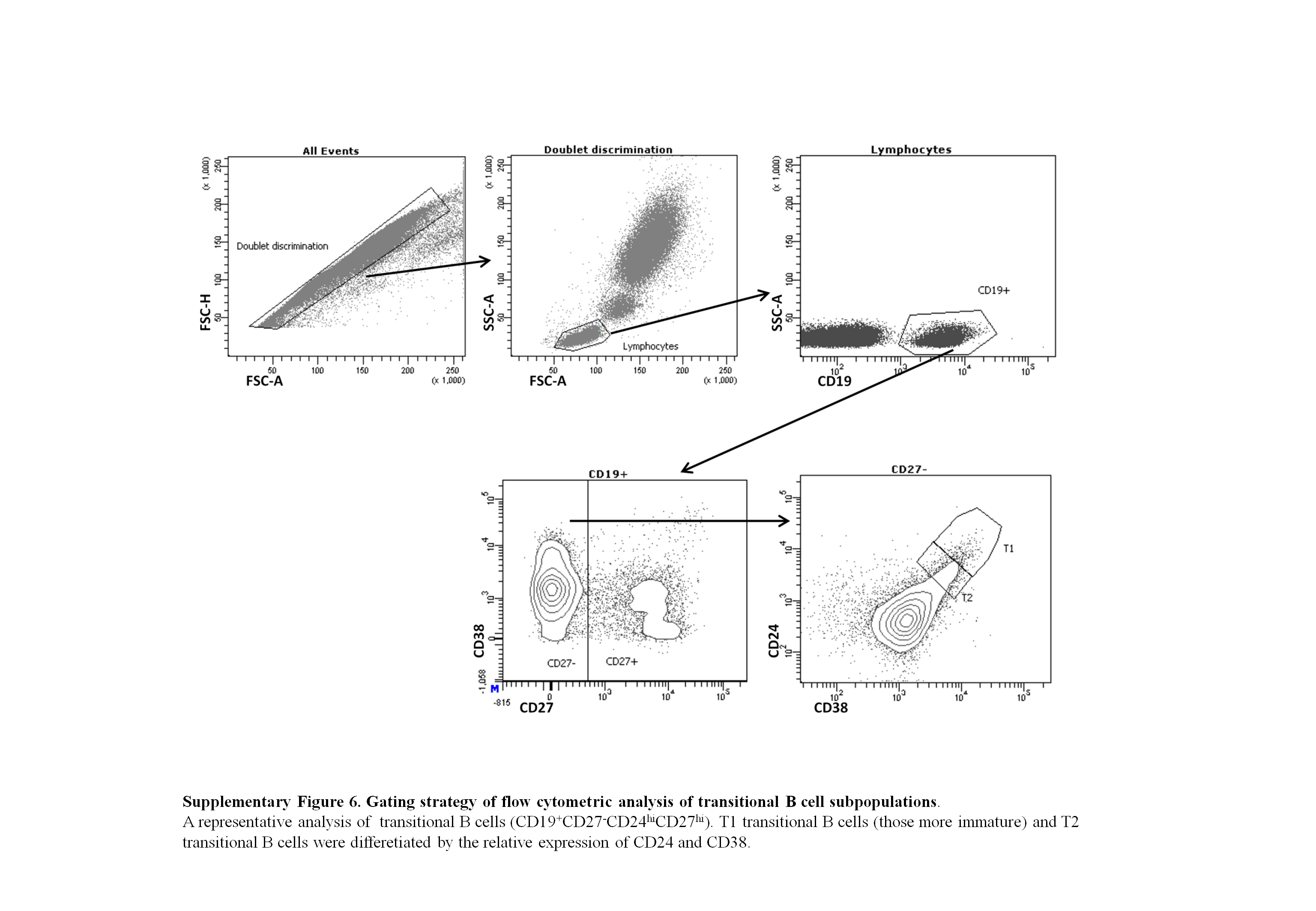

Supplement: Supplementary file 6 [file Image_6.tif]

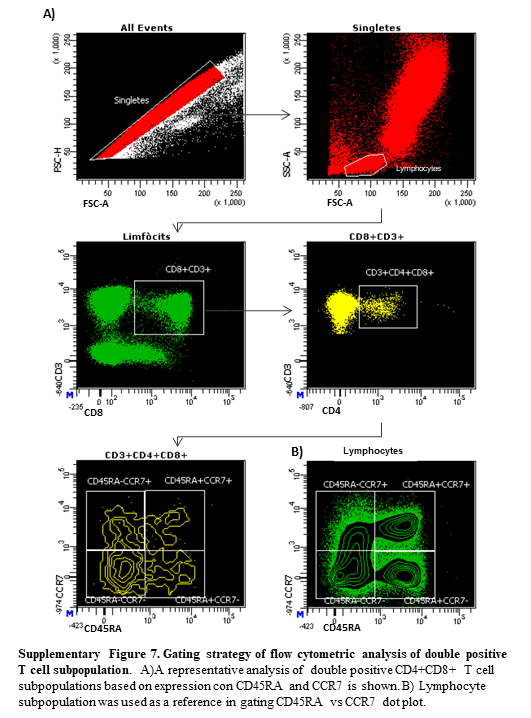

Supplement: Supplementary file 7 [file Image_7.tif]
