# Supplementary material for: Identifying Changes in Peripheral Lymphocyte Subpopulations in Adult Onset Type 1 Diabetes
Source: Front Immunol. 2021 Dec 6;12:784110. doi: 10.3389/fimmu.2021.784110 (PMC8685245; doi:10.3389/fimmu.2021.784110)
Supplement: Supplementary file 8 [file Table_1.docx]

Supplementary Material

**Supplementary table 1. Characteristics of type 1 diabetes patients**

| **Code** | **Gender** | **Age at onset (years)** | **Months from onset** | **AntiGAD Ab** | **IA-2 Ab** | **ICAS** | **anti-insulin Ab** |
| --- | --- | --- | --- | --- | --- | --- | --- |
|  |  |  |  | **Ref Values 0-1 U/ml** | **Ref Values 0,1-14,9u/mL** | **Ref Values < 2 Un. JDF** | **Ref Values 0-8,5 %** |
| T1D_01 | F | 30 | 3 | 20.4 | ND | ND | ND |
| T1D_02 | F | 27 | 4 | 9.6 | ND | <2 | ND |
| T1D_03 | M | 16 | 4 | 4.4 | ND | <2 | ND |
| T1D_04 | M | 30 | 2 | 8 | ND | ND | ND |
| T1D_05 | F | 40 | 3 | >250 | ND | ND | ND |
| T1D_06 | F | 15 | 5 | 237 | ND | <2 | 5.4 |
| T1D_07 | M | 21 | 4 | >250 | ND | <2 | 5.3 |
| T1D_08 | M | 23 | 1 | 4.6 | <1 | <2 | 3.6 |
| T1D_09 | M | 40 | 2 | >250 | ND | ND | ND |
| T1D_10 | M | 46 | 1 | 112 | ND | ND | ND |
| T1D_11 | M | 29 | 2 | 119 | ND | ND | ND |
| T1D_12 | M | 20 | 3 | >250 | ND | ND | ND |
| T1D_13 | M | 37 | 1 | >250 | ND | <2 | 2.8 |
| T1D_14 | M | 33 | 1 | 76 | >400 | ND | ND |
| T1D_15 | F | 34 | 3 | >250 | ND | ND | ND |
| T1D_16 | M | 20 | 3 | ND | ND | ND | ND |
| T1D_17 | M | 31 | 4 | 166 | ND | ND | ND |
| T1D_18 | M | 21 | 2 | 33 | ND | <2 | 4 |
| T1D_19 | F | 24 | 3 | >250 | ND | ND | ND |
| T1D_20 | F | 27 | 2 | >250 | ND | ND | ND |
| T1D_21 | F | 39 | 4 | 269 | ND | ND | ND |
| T1D_22 | F | 25 | 4 | >250 | ND | <2 | <1 |
| T1D_23 | F | 42 | 4 | >250 | 144 | Positive | 6 |
| T1D_24 | F | 18 | 3 | 245 | ND | Positive | ND |
| T1D_25 | F | 27 | 2 | 29 | <7,5 | ND | ND |
| T1D_26 | M | 33 | 2 | >100 | ND | ND | 5 |
| T1D_27 | M | 22 | 5 | 3.1 | 2.3 | <2 | Negative |
| T1D_28 | M | 42 | 5 | >100 | <7,5 | ND | 4.5 |
| T1D_29 | F | 25 | 1 | <0,1 | <7,5 | ND | 4.8 |
| T1D_30 | F | 23 | 1 | >100 | >100 | 64 | ND |
| T1D_31 | F | 31 | 5 | 2.9 | ND | ND | Negative |
| T1D_32 | M | 19 | 4 | 22.7 | >100 | ND | 6.3 |
| T1D_33 | F | 38 | 3 | >100 | 75.3 | ND | ND |
| T1D_34 | F | 34 | 6 | >100 | ND | ND | 6.2 |
| T1D_35 | M | 26 | 10 | > 100 | ND | ND | 8.6 |
| T1D_36 | M | 22 | 1 | 0.48 | ND | ND | 2.69 |
